# Complete Genome Sequence of the Methicillin-Resistant *Staphylococcus aureus* Colonizing Strain M92

**DOI:** 10.1128/genomeA.00478-17

**Published:** 2017-06-08

**Authors:** Jo-Ann McClure, Kunyan Zhang

**Affiliations:** a Centre for Antimicrobial Resistance, Alberta Health Services/Calgary Laboratory Services/University of Calgary, Calgary, Alberta, Canada; b Department of Pathology & Laboratory Medicine, University of Calgary, Calgary, Alberta, Canada; c Department of Microbiology, Immunology and Infectious Diseases, University of Calgary, Calgary, Alberta, Canada; d Department of Medicine, University of Calgary, Calgary, Alberta, Canada; e The Calvin, Phoebe and Joan Snyder Institute for Chronic Diseases, University of Calgary, Calgary, Alberta, Canada

## Abstract

M92 is a methicillin-resistant *Staphylococcus aureus* (MRSA) colonizing strain belonging to ST239-MRSA-III. It frequently shows local nasal colonization in our hospital staff, but has never been associated with infection. We sequenced the complete genome of M92, in order to compare it to highly virulent MRSA strains to gain insight into MRSA virulence factors.

## GENOME ANNOUNCEMENT

*Staphylococcus aureus* is a major human pathogen causing a wide variety of illnesses, ranging from mild skin and soft tissue infections to life-threatening illnesses such as septicemia, endocarditis, and hemorrhagic pneumonia (1). Methicillin-resistant *Staphylococcus aureus* (MRSA) continues to be a major cause of both hospital-associated (HA-MRSA) and community-associated (CA-MRSA) infection, prompting studies to characterize and understand its virulence mechanism (2, 3). MRSA colonizing strain M92 was found and isolated in our local hospitals in the Calgary, Canada area in the 1980s. This strain is frequently showing local nasal colonization in our hospital staff, but has never been associated with infection over the course of many years. M92 belongs to ST239-MRSA-III with accessory gene regulator (agr) type I. The staphylococcal protein A gene (*spa*) is truncated in M92. It has been used as an avirulence control strain in many of our infection models (4–6). We sequenced the complete genome of M92, in order to compare it to those highly virulent MRSA strains to give a more complete understanding of MRSA virulence factors.

The M92 genome was sequenced with Pacific Biosciences (PacBio) RSII sequencing technology, using one single-molecule real-time (SMRT) cell. Raw reads numbering 21,177 were generated covering a total of 239,169,848 sequenced bases, with an average read length of 11,293 bp (longest 48,331 bp). Contig assembly was done using the HGAP workflow (7): short subreads were aligned to the long subreads in a preassembly step using BLASR (8), then the corrected long reads were used to generate contigs with Celera Assembler (9). The contigs were polished by aligning raw reads on them with BLASR, then processed in the variant calling algorithm (Quiver) to generate consensus sequences. The resultant genome was determined to be 3,051,263 bp in length, with an estimated genome coverage of 77× and G+C content of 32.80%. Gene annotation was done using NCBI’s Prokaryotic Genomes Annotation Pipeline, identifying a total of 3,152 genes, of which 3,009 were coding sequences (CDS), 83 were RNA genes, and 60 were pseudogenes.

Sequence analysis indicates that both the genome size and G+C content are in line with other *S. aureus* genomes, however, the size is on the higher end of what has been published. Several virulence factors common in staphylococci were located, including genes for adhesins like fibrinogen binding (*clfA*, *sdrC*, *sdrD*, *sdrE*), fibronectin binding (*fnbA*, *fnbB*), collagen binding (*cna*), elastin binding (*ebpS*), intercellular adhesion (*ica*), and the MHCII analogue (*map*). Common enterotoxin genes were missing (*sea*, *seb*, sec, *sed*, see, *seg*, *seh*, *sei*, *sej*), as was the toxic shock protein gene (*tst*) and immunity evasive gene (*chp*). Genes for enterotoxins *sek* and *seq* were present, as was the immunity evasive gene *scn*, and cytotoxin genes *hla*, *hld*, and *hlg*. Genes for exoenzymes such as the protease *v8*, hyaluronate lyase (*hysA*), and staphylokinase (*sak*) were also found. A more complete analysis is currently underway looking at a broader range of virulence factors, as well as comparing them to those found in more virulent strains of MRSA.

### Accession number(s).

The genome sequence has been deposited at DDBJ/EMBL/GenBank under accession number CP015447.
